# Review of the Association Between Long-Term and Current Systemic Steroid Use With Electromechanical Complications and Inpatient Mortality After ST-Elevation Myocardial Infarction

**DOI:** 10.7759/cureus.55154

**Published:** 2024-02-28

**Authors:** Dennis D Kumi, Rohan Gajjar, Joshua T Narh, Edwin Gwira-Tamattey, Muhammad Sana, Nana Yaa Ampaw, Anna Oduro, Samuel M Odoi, Sheriff Dodoo, Setri Fugar

**Affiliations:** 1 Internal Medicine, John H. Stroger, Jr. Hospital of Cook County, Chicago, USA; 2 Cardiology, Maimonides Medical Center, New York, USA; 3 Internal Medicine, John H. Stroger, Jr. Hospital of Cook County, chicago, USA; 4 Emergency Medicine, Korle-Bu Teaching Hospital, Accra, GHA; 5 Medicine, Kreiskrankenhaus Bergstraße GmbH, Heppenheim, DEU; 6 Cardiology, Northeast Georgia Medical Center Gainesville, Gainesville, USA; 7 Cardiology, Medical College of Wisconsin, Milwaukee, USA

**Keywords:** implantable cardioverter-defibrillator, ventricular septal defect, long-term (current) systemic steroids, electrical dysfunction, mechanical myocardial complications, thrombotic complications, hemodynamic dysfunction, post-mi complications, long-term systemic steroid use, st-elevation myocardial infarction

## Abstract

Background

The impact of long-term systemic steroid use on electrical and mechanical complications following ST-segment elevation myocardial infarction (STEMI) has not been extensively studied.

Methods

In a retrospective cohort study of the National Inpatient Sample (NIS) from 2018 to 2020, adults admitted with STEMI were dichotomized based on the presence of long-term (current) systemic steroid (LTCSS) use. The primary outcome was all-cause mortality. Secondary outcomes included a composite of mechanical complications, electrical, hemodynamic, and thrombotic complications, as well as revascularization complexity, length of stay (LOS), and total charge. Multivariate linear and logistic regressions were used to adjust for confounders.

Results

Out of 608,210 admissions for STEMI, 5,310 (0.9%) had LTCSS use. There was no significant difference in the odds of all-cause mortality (aOR: 0.89, 95%CI: 0.74-1.08, p-value: 0.245) and the composite of mechanical complications (aOR: 0.74, 95%CI: 0.25-2.30, p-value: 0.599). LTCSS use was associated with lower odds of ventricular tachycardia, atrioventricular blocks, new permanent-pacemaker insertion, cardiogenic shock, the need for mechanical circulatory support, mechanical ventilation, cardioversion, a reduced LOS by 1 day, and a reduced total charge by 34,512 USD (all p-values: <0.05). There were no significant differences in the revascularization strategy (coronary artery bypass graft (CABG) vs. percutaneous coronary interventions (PCI)) or in the incidence of composite thrombotic events.

Conclusion

LTCSS use among patients admitted with STEMI was associated with lower odds of electrical dysfunction and hemodynamic instability but no difference in the odds of mechanical complications, CABG rate, all-cause mortality, cardiac arrest, or thrombotic complications. Further prospective studies are needed to evaluate these findings further.

## Introduction

Glucocorticoids have become common anti-inflammatory agents for several treatment indications. Despite their efficacy and better short-term safety profile, they are fraught with metabolic side effects, such as impaired glycemic control, deranged lipid metabolism, and hypothalamic-pituitary-adrenal (HPA) axis suppression [1]. These can all lead to unfavorable cardiovascular outcomes in the medium to long term. Roubille C et al. performed a meta-analysis of 34 studies in patients with rheumatoid arthritis. They noted that steroid use was associated with an increased risk of all cardiovascular events, including myocardial infarction (MI), stroke, and heart failure [2].

Growing evidence supports the detrimental role of unchecked post-MI systemic inflammatory response, which often leads to adverse outcomes [3]. Experimental evidence has shown that the use of high-dose steroids after MI may reduce infarct size, thus preserving ejection fraction and possibly reducing the rate of cardiogenic shock and mortality [4,5]. Empirical evidence in favor of or against the effect of long-term steroid use on MI is conflicting. Several studies have linked steroid therapy and high serum cortisol to the occurrence of major adverse cardiac events in the medium to long term [6-8]. On the other hand, other studies have either found no difference or identified a somewhat mitigating effect of steroids on the incidence and outcomes after acute MI [9-11].

In current cardiovascular medicine practice, glucocorticoids are indicated in conditions such as myocarditis, persistent pericarditis, and Dressler’s syndrome. The use of anti-inflammatory agents such as colchicine after acute MI has shown promise [12,13]. The use of steroids in patients with acute MI has been limited due to concerns about free wall rupture and other mechanical complications due to poor wound healing [14]. This risk has, however, not been identified in other studies [11].

Existing literature has studied the impact of a short-term trial of steroids after acute myocardial infarction with contrasting reports. Not much is known about the impact of long-term, including current, systemic steroid use on the inpatient outcomes after ST-segment elevation myocardial infarction (STEMI). Due to the paucity of satisfactory evidence, we sought to use large national databases to analyze the outcomes of mechanical and electrophysiological complications of STEMI in patients receiving long-term systemic steroid therapy and the subsequent hemodynamic effects.

## Materials and methods

Study design and data source

A retrospective cohort study with data from the combined National Inpatient Sample (NIS) database from 2018 to 2020 was employed. Using the International Classification of Diseases, Tenth Revision (ICD-10) codes, adult patients admitted with a primary diagnosis of STEMI were dichotomized based on the presence of a secondary diagnosis of long-term (current) systemic steroid (LTCSS) use. We studied baseline patients’ and hospital-level characteristics and assessed various primary and secondary outcomes. The NIS is a large administrative database provided by the Agency for Healthcare Research and Quality (AHRQ), which contains data from approximately a 20% sample of inpatient hospitalizations in the United States. The NIS includes information on the principal diagnosis, which is the main reason for hospitalization, identified by the primary ICD-10 code, as well as secondary diagnoses recorded during the hospitalization. This database provides a valuable resource for researchers to examine patterns of care and outcomes in a nationally representative sample of hospitalized patients in the US [15]. Relevant ICD-10 codes are provided in Appendix 1.

Patient population and outcomes of the study

Our study population included all adult patients (≥18 years of age) who were hospitalized over the study period. We sampled patients admitted for STEMI and analyzed patient characteristics, such as demographics, hospital-level characteristics, and relevant medical comorbidities. We identified involved culprit coronary vessels, including the left main artery, left anterior descending artery (LAD), left circumflex artery, and right coronary artery. The primary outcome was all-cause mortality. Secondary outcomes were a composite of mechanical complications (presence of post-MI ventricular free wall rupture, post-MI ventricular septal defect, post-MI ventricular aneurysm, post-MI papillary muscle rupture, or post-MI hemopericardium), electrophysiological complications (atrioventricular block, new permanent pacemaker insertion, cardioversion rate, new ICD insertion), composite thrombotic event (acute venous thromboembolic events and intracardiac thrombus), and hemodynamic complications (cardiac arrest, cardiogenic shock, septic shock, rate of mechanical circulatory support, and mechanical ventilation). We also assessed revascularization complexity, including percutaneous coronary interventions (PCI), multi-stent PCI (2 or more stents), and coronary artery bypass graft (CABG), as well as length of stay (LOS) and total charge.

Statistical analysis

We performed statistical analyses using Stata, version 17 (Stata Corp, College Station, TX) standard edition. The Whitney-Mann U test for non-parametric data was used to compare group differences in continuous variables, while group differences in categorical variables were compared using Pearson’s chi-square analysis and Fisher's exact test. Univariate logistic and linear regression analysis was conducted using all available variables and comorbidities to calculate unadjusted odds ratios for the study outcomes. We included all variables with p-values less than 0.1 in a multivariate logistic and linear regression model to calculate the odds ratios of our study outcomes while controlling for significant confounders. All tests were double-sided. We considered outcomes with p-values less than 0.05 and a 95% confidence interval to be statistically significant. To adjust for the comorbidity burden, we used the Charlson Comorbidity Index.

Ethical considerations

All patient data in the NIS are de-identified, and the data is publicly available. Therefore, we did not seek institutional review board approval for this study.

## Results

Baseline characteristics

Over the study period, there were 608,210 admissions for STEMI, including 5,310 (0.9%) with LTCSS use. Patients with LTCSS use were 4 years older (68 years vs. 64 years, p-value <0.001), included a higher proportion of females (41.53% vs. 31.52%), were predominantly white, and had a higher prevalence of most comorbidities, including interstitial lung disease, sarcoidosis, rheumatological disorders (systemic lupus erythematosus (SLE), rheumatoid arthritis, systemic sclerosis, psoriasis, dermatomyositis, polymyositis, and antineutrophilic cytoplasmic antibody (ANCA)-vasculitis), chronic kidney disease (CKD), chronic obstructive pulmonary disease (COPD), asthma, lymphoid and myeloid malignancy, hypothyroidism, and inflammatory bowel disease (all p-values <0.05). Hospital-level characteristics, including hospital size and teaching status, were similar between the groups. Details of baseline characteristics and univariate regression analysis are summarized in Table 1.

**Table 1:** Baseline population characteristics and culprit coronary artery among patients admitted with ST-elevation myocardial infarction with or without long-term (current) systemic steroid use. **Statistically significant p-value <0.05. LTCSS: Long-term (current) systemic steroid; STEMI: ST-elevation myocardial infarction; Composite Mechanical Complications: Post-myocardial infarction (MI) ventricular free wall rupture, post-MI ventricular septal defect, post-MI papillary muscle rupture, post-MI hemopericardium, and post-MI left ventricular apical aneurysm; PEM: Protein-energy malnutrition; CKD: Chronic kidney disease; COPD: Chronic obstructive pulmonary disease; IBD: Inflammatory bowel disease; ILD: Interstitial lung disease.

Baseline characteristics	STEMI without LTCSS use (n= 602,900)	STEMI with LTCSS use (n = 5,310)	P-value **	
				Demography and hospital-level characteristics				
Age/years	64	68	<0.001	
Sex (Male)	68.48%	58.47%	<0.001	
Race			<0.001	
White	74.48%	81.63%		
Black	9.43%	7.97%		
Hispanic	8.84%	5.73%		
Asian/pacific islander	3.02%	1.65%		
Native American	0.58%	0.39%		
Other	3.64%	2.62%		
Charlson comorbidity index			<0.001	
0-1	28.00%	11.49%		
2	29.16%	25.24%		
3 or more	42.84%	63.28%		
Expected primary payer			<0.001	
Medicare	49.11%	66.28%		
Medicaid	11.16%	6.99%		
Private insurance	32.91%	23.28%		
Self-pay and others	6.82%	3.45%		
Hospital bed size			0.115	
Small	17.12%	17.80%		
Medium	29.60%	31.11%		
Large	53.28%	50.09%		
Hospital location and teaching status			0.373	
Rural	6.53%	7.63%		
Urban non-teaching	18.41%	18.08%		
Urban teaching	75.06%	74.29%		
Patient comorbidities				
Alcohol use disorder	3.6%	2.07%	0.007	
Coronary artery disease	12.21%	9.51%	0.007	
Heart failure	7.60%	8.29%	0.407	
Stroke history	0.63%	0.38%	0.298	
Diabetes Mellitus	34.08%	33.05%	0.482	
Hypertension	42.28%	39.83%	0.104	
CKD	15.62%	19.87%	<0.001	
Hypothyroidism	9.31%	15.25%	<0.001	
Obesity	18.39%	17.23%	0.330	
PEM	3.36%	3.86%	0.390	
COPD	10.52%	21.85%	<0.001	
Asthma	3.51%	6.97%	<0.001	
Lymphoid and myeloid malignancy	0.87%	1.51%	0.027	
ILD	0.17%	0.94%	<0.001	
IBD	0.96%	2.64%	<0.001	
Sarcoidosis	0.16%	1.41%	<0.001	
HIV	2.59%	1.88%	0.150	
Rheumatologic disorder	1.94%	29.47%	<0.001	
Culprit coronary vessel				
Left main coronary artery	1.43%	1.68%	0.924	
Left anterior descending artery	46.51%	40.76%	0.139	
Left circumflex artery	9.46%	9.24%	0.782	
Right coronary artery	42.60%	48.42%	0.214	

**Figure 1:**
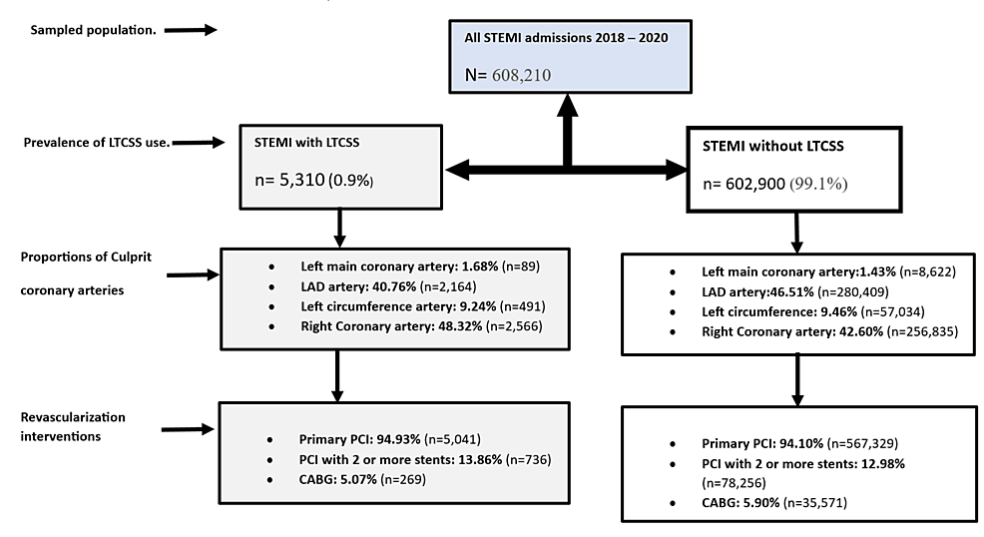
Flow diagram of all admissions for STEMI with and without long-term (current) systemic steroid use and revascularization interventions. STEMI: ST-elevation myocardial infarction; LTCSS: Long-term (current) systemic steroids; LAD: Left anterior descending; PCI: Percutaneous coronary intervention; CABG: Coronary artery bypass graft.

Additionally, there were no significant differences in the types of involved culprit coronary arteries identified and in the pattern of revascularization complexity between the two cohorts, as depicted in Figure 1.

Primary and secondary outcomes

Details of clinical outcomes and results of multivariable regression analysis of outcome measures are summarized in Table 2.

**Table 2:** Adverse clinical outcomes among patients admitted with ST-elevation myocardial infarction based on their use of long-term (current) systemic steroids. Composite Mechanical Complications: Post-myocardial infarction (MI) ventricular free wall rupture, post-MI ventricular septal defect, post-MI papillary muscle rupture, post-MI hemopericardium, and post-MI left ventricular apical aneurysm; CABG: Coronary artery bypass graft; PCI: Percutaneous coronary intervention; Multi-Stent PCI: Involving 2 or more coronary stents; AV: Atrioventricular; ICD: Implantable cardioverter defibrillator; VTE: Venous thromboembolic events. *Adjustments were made for baseline characteristics, including age, sex, race, hospital bed size, coronary artery disease, hypertension, heart failure, chronic kidney disease, diabetes mellitus, stroke, hypothyroidism, obesity, protein-energy malnutrition, asthma, COPD, myeloid and lymphoid malignancy, interstitial lung disease, inflammatory bowel disease, and HIV. **Statistically significant p-value <0.05.

OUTCOMES	STEMI with long-term (current) systemic steroid use	STEMI without long-term (current) systemic steroid use	Adjusted Odds ratio* Adjusted difference* (95% Confidence interval)	**P-value
Primary Outcome				
All-cause mortality	10.46% (n=555)	11.86% (n=71,504)	0.89 (0.74-1.08)	0.254
Secondary Outcomes				
Revascularization strategy complexity				
Primary PCI	94.93% (n=5,041)	94.10% (n=567,329)	1.16 (1.02-1.32)	0.027
Multi-stent PCI	13.86% (n=736)	12.98% (n=78,256)	1.07 (0.84-1.35)	0.584
CABG rate	5.07% (n=269)	5.90% (n=35,571)	0.84 (0.59-1.19)	0.324
Mechanical complications				
Composite Mechanical complications	0.28% (n=15)	0.33% (n=1,990)	0.74 (0.25-2.30)	0.599
Electrophysiologic complications				
Ventricular tachycardia	8.66% (n=460)	13.39% (n=80,728)	0.63 (0.51-0.78)	<0.001
Cardioversion	3.01% (n=160)	4.37% (n=26347)	0.67 (0.47-0.95)	0.023
Second- and third-degree AV block	3.86% (n=205)	4.93% (n=29,723)	0.69 (0.50-0.94)	0.018
New permanent pacemaker	2.07% (n=110)	3.38% (n=20,378)	0.57 (0.37-0.86)	0.008
New ICD insertion	0.47% (n=25)	0.86% (n=5,185)	0.52 (0.22-1.26)	0.148
Hemodynamic instability				
Cardiac arrest	6.21% (n=330)	6.75% (n=40,696)	0.87 (0.68-1.12)	0.288
Cardiogenic shock	13.28% (n=705)	14.78% (n=89,109)	0.77 (0.64-0.92)	0.004
Mechanical circulatory support device	8.00% (n=425)	10.41% (n=62,762)	0.71 (0.56-0.89)	0.003
Mechanical ventilation	11.30% (n=600)	13.86% (n=83,562)	0.68 (0.56-0.83)	<0.001
Septic shock	3.36% (n=178)	3.18% (n=19,172)	0.88 (0.63-1.23)	0.461
Thrombotic events				
Composite thrombotic event	2.07% (n=110)	1.59% (n=9,586)	1.15 (0.75-1.76)	0.525
Healthcare Utilization				
Length of stay/days	4	5	-1 (-0.61 to -1.16)	<0.001
Total Charge/ USD	107,102.8	131,310.6	-28,110.73 (-34,511.6 to -21709.89)	<0.001

There was no significant difference in all-cause mortality (aOR: 0.89, p-value: 0.245), composite mechanical complications (aOR: 0.74, p-value: 0.599), CABG, or PCI rates between STEMI patients with LTCSS use compared with those without. LTCSS use was associated with lower odds of most electrophysiological complications, including lower odds of ventricular tachycardia (aOR: 0.63, p-value: <0.001), atrioventricular blocks (aOR: 0.69, p-value: 0.018), new permanent pacemaker insertion (aOR: 0.57, p-value: 0.008), and rate of cardioversions (aOR: 0.67, p-value: 0.023). Except for higher odds of adrenal crisis (aOR: 15.76, p-value: <0.001), LTCSS use was associated with lower odds of hemodynamic instability, including lower odds of cardiogenic shock (aOR: 0.77, p-value: 0.004), need for mechanical circulatory support (aOR: 0.71, p-value: 0.003), and mechanical ventilation (aOR: 0.68, p-value: <0.001), without a difference in odds of cardiac arrest, new ICD insertions, or septic shock. There was no difference in thrombotic complications such as intracardiac thrombi or acute venous thromboembolic events between the two cohorts. With regards to health care resource utilization, there was also a reduction in length of stay by 1 day (p-value <0.001) and total charge by 34,512 USD (p-value: <0.001) among patients with LTCSS.

**Figure 2:**
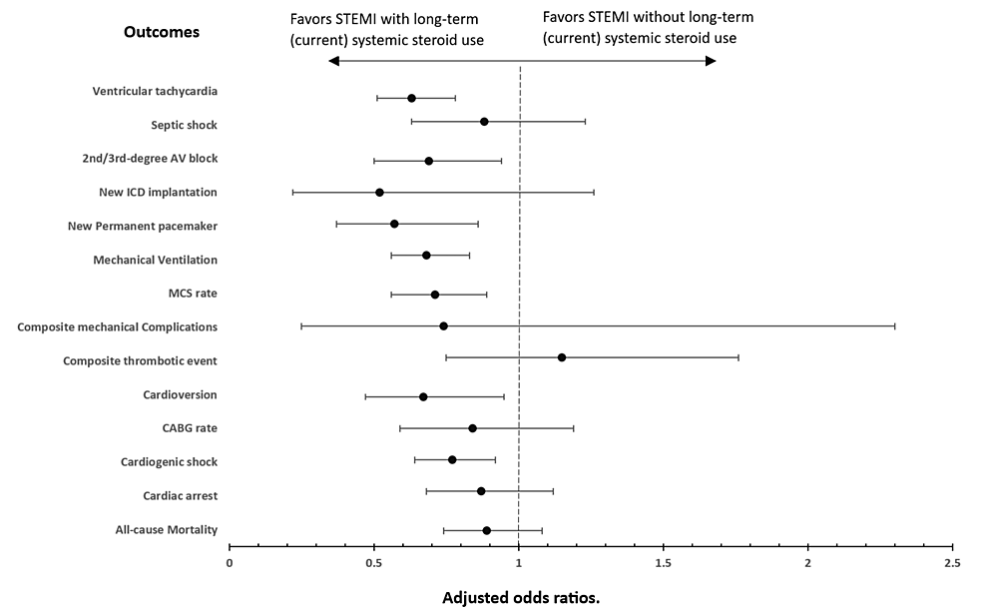
Plot of odds ratios for electrical, mechanical, hemodynamic, and thrombotic complications, including all-cause mortality, among patients admitted with STEMI who had long-term (current) systemic steroid use. STEMI: ST-elevation myocardial infarction; Mechanical Complications: A composite of post-STEMI mechanical complications, including post-myocardial infarction (MI) ventricular free wall rupture, post-MI ventricular septal defect, post-MI papillary muscle rupture, post-MI hemopericardium, and post-MI left ventricular apical aneurysm; 2nd/3rd-degree AV block: Second-degree or third-degree atrioventricular blocks; ICD: Implantable cardioverter defibrillator; MCS: Mechanical circulatory support; CABG: Coronary artery bypass graft; COPD: Chronic obstructive pulmonary disease. *Odds ratios are adjusted for baseline characteristics, including age, sex, race, hospital bed size, coronary artery disease, hypertension, heart failure, chronic kidney disease, diabetes mellitus, stroke, hypothyroidism, obesity, protein-energy malnutrition, asthma, COPD, myeloid and lymphoid malignancy, interstitial lung disease, inflammatory bowel disease, and HIV.

A graphical presentation of odds ratios of primary and secondary outcomes based on the presence or absence of LTCSS use among STEMI patients is depicted in Figure 2.

## Discussion

There is no consensus regarding the impact of LTCSS on outcomes among patients presenting with STEMI [6-11]. Medium to long-term steroid use has been associated with an increased risk of adverse cardiovascular outcomes; however, there are limited studies focused on the in-hospital outcomes of patients presenting with STEMI. In this study, utilizing a NIS database, there was no significant difference in hospital mortality or mechanical complications among patients on LTCSS therapy compared to those without. Patients on LTCSS had an overall lower incidence of electrophysiologic and hemodynamic complications, and overall, there was a decrease in healthcare resource utilization compared to patients not on LTCSS.

Baseline characteristics

From our study, we estimate the prevalence of LTCSS use among inpatient STEMI populations in the United States to be 0.9%. Data on LTCSS use, often defined as usage exceeding three months, are limited in the U.S. One study estimated steroid use in the United States at 1.2% using the National Health and Nutrition Examination Survey (NHANES) data [16]. A United Kingdom study, which looked at a 20-year trend analysis, estimated glucocorticoid use to be 0.85% [17]. Our study revealed a male predominance in the entire cohort of patients presenting with STEMI, in line with prevalence data for STEMI; however, there was a 10% higher representation of females in the LTCSS use cohort. This is in keeping with the higher prevalence of chronic inflammatory or autoimmune conditions among females and the higher overall use of LTCSS among females [18]. The older age of the collated cohort may also reflect the later presentation of atherosclerotic events at a post-menopausal age in females. A higher prevalence of the white population is unclear and an area worth looking into. Based on the weathering hypothesis, many chronic inflammatory conditions have been projected to be prevalent among non-white populations [19]. Data on racial estimates for LTCSS use and what may account for any disparities are lacking. We believe that the white predominance may reflect a general pattern of racial distribution of STEMI admissions in the U.S. [20]. Overall, patients on LTCSS admitted with STEMI had a higher prevalence of chronic inflammatory conditions. In a study by Boukhris M et al. reviewing the SCALIM registry, the prevalence of chronic inflammatory disorders among patients admitted with STEMI was estimated at 5% [6].

Mechanical and electrophysiological complications

In current practice, expedited emergency medical responses leading to shorter door-to-balloon times have significantly reduced STEMI-related mortality except in patients with cardiogenic shock, malignant arrhythmias, heart failure, or mechanical complications. From our study, LTCSS was not associated with increased odds of post-STEMI composite mechanical complications, contrary to prevailing hypotheses. The traditional belief of increased free wall rupture and other mechanical complications is based on the concept of delayed wound healing, often in surgical patients. Empirical data on this, however, have been conflicting. A meta-analysis of the use of steroids in acute myocardial infarction by Giugliano et al. showed no difference in free-wall rupture [11]. Similarly, in a study conducted by Contoli M et al. between 2003 and 2009 looking at outcomes of STEMI in nearly 2000 COPD patients, a lower rate of cardiogenic shock and pulmonary edema was found [10]. Electrophysiological complications, especially unstable ventricular tachycardia, and other malignant arrhythmias, are among the causes of early death in the post-STEMI period. From our study, LTCSS was associated with significantly lower odds of ventricular tachycardia. Halonen J et al. found that patients undergoing CABG who had postoperative atrial fibrillation had higher inflammatory markers and that administration of steroids reduced this incidence significantly [21]. Stress-induced dysregulated adrenergic response, ischemia-associated reentry pathways, calcium signaling abnormalities leading to after-depolarizations, and the effects of inotropes and vasopressors are possible triggers for electrical dysfunction. In an animal model, administration of steroids led to reduced intracellular calcium signaling and mitochondria injury and was associated with a reduced incidence of arrhythmia [22]. We also observed a lower odd of cardioversion among patients with LTCSS, thus suggesting that even among those with some arrhythmia, there was either more spontaneous conversion or they were less hemodynamically significant. Additionally, from our study, we observed lower odds of atrioventricular blocks (second-degree and third-degree AV block) and the need for new ICD placement among patients on LTCSS. Lown B et al. identified the beneficial effects of steroids in enhancing P-R conduction, and this could explain the protective effect against post-MI functional heart blocks [23]. 

Hemodynamic instability and death

From our study, there were significantly lower odds of hemodynamic complications including cardiogenic shock, the need for a mechanical circulatory device, and mechanical ventilation. This was despite having similar culprit vessels and complexities of revascularization interventions between the two cohorts. Data on the effect of steroid use on hemodynamic outcomes in cardiac patients have been well-studied among patients undergoing cardiorespiratory bypass. For instance, Whitlock RP et al., from a randomized controlled trial assessing the effect of pulsed dose steroids in patients undergoing cardiopulmonary bypass, reported lower rates of systemic inflammatory response syndrome (SIRS) and elevated serum inflammatory markers, shorter ICU stays, lower need for vasopressor therapy, and lower incidence of arrhythmias among patients treated with steroids [24]. Similarly, Doolub G et al. queried the NIS database between 2015 and 2018 to evaluate outcomes of PCI in patients with acquired immunosuppression and found lower in-hospital mortality, cardiogenic shock, cardiac arrhythmia, and vascular complications among patients with steroids compared to others [25]. Consequently, we noted that despite the higher overall comorbidity and age in patients with LTCSS use, there was a trend towards lower mortality even after adjusting for age, sex, and comorbidities. This is likely a reflection of lower hemodynamic instability, lower electrophysiological complications, and a trend towards lower mechanical complications balanced by the overall higher frailty with aging and end-organ complications from increased comorbid conditions. An earlier meta-analysis by Barzilai D et al. showed a mortality reduction with hydrocortisone use in acute MI patients [26]. In another meta-analysis, the investigators noted that after sensitivity analysis, there was an insignificant trend towards lower mortality after acute myocardial infarction among patients who received steroids [11]. Similarly, another study examining COPD patients on long-term inhaled steroids admitted with STEMI also found lower short-term mortality [10]. Tavakoli N et al. also demonstrated that hydrocortisone had favorable neurologic outcomes even after cardiogenic shock [27].

Thromboembolic complications

Finally, steroids have been associated with increased thrombotic risk by increasing prothrombotic factors including p-selectin and von Willebrand factor in serum [28,29]. Data on arterial thrombosis including post-MI left ventricular thrombus are not well studied. Our study thus looked at post-STEMI composite thrombotic events and found no difference between the two cohorts but a trend toward an increased association. These favorable findings were likely not impacted by hospital-level characteristics as there were no differences in hospital size, teaching status, or urban location among the studied population.

Biologic targets associated with post-STEMI complications and effect of steroid therapy

In our bid to provide a biologic basis for the observations, our extensive literature search uncovered three main theories: local reperfusion injury, an extensive systemic inflammatory response, and the detrimental effects of hyperaldosteronism as biologic pathways that could be mitigated to reduce morbidity and mortality after STEMI. We believe that the modulating effects of long-term steroids on these targets may offer some explanations. After a myocardial infarction, an interplay of adequate vs. exuberant inflammatory response is key to survival. Post-MI reperfusion injury has been an area of concern as it has been associated with the determination of final infarct size, persistently low ejection fraction, and other adverse outcomes [30]. Damage-associated molecular patterns (DAMPs) are released from necrosed myocardium which initiate a cascade of nonspecific immunologic reactions. Initially, there is recruitment of leukocytes into injured myocardium based on chemokines and complement-mediated inflammatory factors [31,32]. This stage is crucial in the repair of injured myocardium but can potentially lead to microvascular dysfunction and eventually myocardial remodeling if unchecked. Following this and under the influence of leukocyte-derived humoral factors, there is a systemic inflammatory response which, in many cases, has been found to be detrimental. Many of the involved inflammatory markers, such as IL-6, CRP, TNF-α, IL-1β, and macrophage inhibitory factor (MIF), have been associated with post-MI hypertrophy, remodeling, persistent systolic dysfunction, heart failure, and mortality [33-37]. Long-term steroid therapy at physiologic doses has anti-inflammatory properties and only minimal immunosuppressive effects. By inhibiting leukocyte margination, steroids potentially reduce this important initial trigger for reperfusion injury [38,39]. Elevated serum aldosterone levels in STEMI patients at presentation have been associated not only with reperfusion injury but also with a higher incidence of atrial fibrillation, all-cause mortality, and cardiac arrest, even after adjustment for age and Killip score [40]. Low-dose chronic steroid use in the physiologic dose range is expected to modestly suppress the aldosterone response and thus attenuate these effects [40].

Study merits and limitations

Our study was based on large, pooled data, which increased its power. The NIS is generated at the hospital level and weighted to reflect the US population, thus, it is a reliable source of patient data to make hypotheses about population-wide problems in the inpatient setting. One limitation is the susceptibility to coding errors, which can affect the quality and reliability of the data. Another limitation is the lack of outpatient data in the NIS. The NIS does not provide information on the onset of symptoms prior to admission, and this could affect the rate of complications. We cannot comment on the dose or duration of steroid use and how that affects clinical outcomes.

## Conclusions

In this US-based study among patients admitted with STEMI, comorbid long-term systemic steroid use was associated with lower odds of electrical dysfunction and hemodynamic instability, but there was no difference in all-cause mortality, mechanical complications, CABG rate, cardiac arrest, or composite thrombotic events. Prospective studies examining the effect of the dose and duration of steroids on post-STEMI mechanical complications would help to expand the current knowledge. Studies looking into ethno-racial differences in the use of steroids and outcomes of atherosclerotic events would be prudent.

## Appendices

Appendix 1 

**Table 3:** Key ICD-10 and PCS codes and corresponding diagnosis and procedures. STEMI: ST-elevation myocardial infarction; MI: Myocardial infarction; ICD: Implantable cardioverter defibrillator; CABG: Coronary artery bypass graft; PCI: Percutaneous coronary intervention; AV: Atrioventricular.

ICD-10 code	Diagnosis/procedure
I21.0, I21.1, I21.2, I 21.3	STEMI
Z79.52	Long term (current) systemic steroid use
I23.2	Post-MI ventricular septal defect
I23.0	Post-MI hemopericardium
I23.5, I23.4	Post-MI papillary muscle rupture
I23.3	Post-MI ventricular free wall rupture
I23.6	Intracardiac thrombus
I47.2	Ventricular tachycardia
02H40KZ, 02H43KZ, 02H44KZ, 02H60KZ, 02H63KZ, 02H64KZ, 02H70KZ, 02H73KZ, 02H74KZ, 02HK0KZ, 02HK3KZ, 02HK4KZ, 02HL0KZ, 02HL3KZ, 02HL4KZ, 02HNOKZ, 02HN3KZ, 02HN4KZ, 0JH608Z, 0JH60FZ, 0JH638Z, 0JH63FZ, 0JH808Z, 0JH838Z, 02H73JZ, 02H70NZ, 02H70JZ, 02H64NZ, 02H64JZ, 02H63NZ, O2H63JZ, 02H60NZ, 02H60JZ	ICD implantation
0JH837Z, 0JH807Z, 0JH637Z, 0JH607Z, 0JH835Z, 0JH805Z, 0JH635Z, 0JH605Z, 02HL4NZ, 02HL4JZ, 02H44NZ, 02HA3RZ, 02H44JZ, 02HL0NZ, 02HL0JZ, 02HK4NZ, 02HK4JZ, 02H43NZ, 02H40JZ, 02HK0NZ, 02HK3JZ, 02HK0JZ, 02H74JZ, 02H73NZ, 02H73JZ, 02H70NZ, 02H70JZ, 02H64NZ, 02H64JZ, 02H63NZ, 02H63JZ, 02H60NZ, 02H60JZ	Permanent pacemaker insertion
I44.1, I44.2	Second-degree or third-degree AV block
5A2204Z	Cardioversion
I462, I469	Cardiac arrest
R570, T8111XA, T8111XS	Cardiogenic shock
0270, 0271, 0272, 0273	PCI
0210, 0212, 0211, 0214	CABG
Z9282, 3E04317, 3E03317	Systemic fibrinolysis
I21.01	STEMI with left main artery involvement
I21.02	STEMI with left anterior descending artery involvement
I21.21	STEMI with left circumflex artery involvement
I21.11	STEMI with right coronary artery involvement
5A02110, 5A02210, 5A02216, 5A02116, 5A0221D, 5A1522G, 5A15223, 5A15A2H, 5A15A2F, 5A1522F, 5A1522H, 5A0211D, 5A15A2G, 02HA3RZ	Mechanical circulatory device
5A1955Z, 5A1935Z, 5A1945Z	Mechanical ventilation
R65.21	Septic shock
